# Evidence for a causal role by human papillomaviruses in prostate cancer – a systematic review

**DOI:** 10.1186/s13027-020-00305-8

**Published:** 2020-07-14

**Authors:** James S. Lawson, Wendy K. Glenn

**Affiliations:** School of Biotechnology and Biomolecular Science, University of New South Wales, Sydney, Australia

**Keywords:** Prostate cancer, Human papillomaviruses, HPV, Transmission, Identification, Prevention

## Abstract

It is hypothesised that high risk for cancer human papillomaviruses (HPVs) have a causal role in prostate cancer.

In 26 case control studies, high risk HPVs have been identified in benign and prostate cancers. High risk HPVs were identified in 325 (22.6%) of 1284 prostate cancers and in 113 (8.6%) of 1313 normal or benign prostate controls (*p* = 0.001).

High risk HPVs of the same type have been identified in both normal and benign prostate tissues prior to the development of HPV positive prostate cancer. High risk HPVs can be associated with inflammatory prostatitis leading to benign prostate hyperplasia and later prostate cancer. Normal human prostate epithelial cells can be immortalised by experimental exposure to HPVs. HPVs are probably sexually transmitted.

The role of HPVs in prostate cancer is complex and differs from HPVs associated cervical cancer. HPV infections may initiate prostate oncogenesis directly and influence oncogenesis indirectly via APOBEC enzymes. HPVs may collaborate with other pathogens in prostate oncogenesis.

Although HPVs are only one of many pathogens that have been identified in prostate cancer, they are the only infectious pathogen which can be prevented by vaccination.

A causal role for HPVs in prostate cancer is highly likely.

## Introduction

We hypothesise that high risk for cancer human papillomaviruses (HPVs) have a causal role in prostate cancer. Although HPVs are only one of many pathogens that have been identified in prostate cancer, they are the only infectious pathogen which can be prevented by vaccination. Hence the importance of assessing the evidence.

We have assessed the evidence by using an extended version of the classic Austin Bradford Hill causal criteria [1]. Hill established the following criteria - strength of association, consistency, specificity, temporality, biological gradient, plausibility, coherence, experiment, and analogy. We have included additional criteria to address more current scientific developments in studies of oncoviruses. These additional criteria include identification of the virus, means of transmission and oncogenic mechanisms. There are differences in the importance of each criterion. With respect to viruses and cancer, the conclusive identification of the virus in cancer tissues and a significant odds ratio between cancer and non-cancer tissues, are of special importance.

## Background

Multiple pathogens including both viruses and bacteria have been identified in prostate cancer tissues [2–5]. These pathogens include human papillomaviruses, Epstein Barr virus and commonly *Propionibacterium acnes*.

There have been several recent meta-analyses of HPVs and prostate cancer by Yang et al., Yin et al., Russo et al. and Moghoofi et al. [6–9]. In both the Russo et al. and Moghoofi et al. studies, data based on both prostate tissues and serum were combined. In our view this is not appropriate because serology identifies overall exposure to HPV infections but does not identify HPV infections of specific organs such as the prostate. The meta-analysis conducted by Yin et al. considered studies which compared the prevalence of high risk HPVs in prostate cancer tissues and benign and normal prostate tissues. The Yin et al. meta-analysis included 24 case control studies involving 971 prostate cancers and 1085 benign and normal prostate controls. There was an increased risk for HPV associated prostate cancer with an odds ratio of 2.27. This outcome was similar to Yang et al. [6]. The extended “Hill” causal criteria, were not considered in these 4 meta-analyses. We have not identified any reviews of the full range of causal criteria with respect to HPVs and prostate cancer.

Reports of the identification of HPVs in prostate cancer have been flowing since 1990/1991 [10]. This was after the advent of PCR, a new scientific method of the time. Contamination has always been a problem and laboratories go to exceptional lengths to avoid it. Not all researchers could find the virus, probably due to the low copy number. The advancement of PCR and identification techniques in recent years have made reports more reliable. HPV has since been identified in 15 countries. In 2006 an alleged new virus, xenotropic murine leukemia virus XMRV, was thought to be involved in prostate cancer. It was later proved to be a laboratory contaminant [11].

There are differences between HPV associated cervical and HPV associated prostate cancers [12]. The HPV viral load is extremely low in prostate cancers as compared to cervical cancer. Cervical cancer mainly involves squamous epithelial cells, prostate cancer mainly involves glandular epithelial cells (although both cancers can have changes in both types of cells).

## Methods

A systematic search of the scientific literature was conducted according to the methods used for the Preferred Reporting Items for Systematic Reviews and Meta-analysis guidelines -PRISMA [13]. We specifically sought to locate studies which (i) identified HPVs in normal, benign and malignant prostate tissues, (ii) assessed the presence of HPV antibodies in serum of normal men and prostate cancer patients, (iii) compared the prevalence of HPVs in normal, benign and prostate cancers (case control studies), (iv) considered the outcome of prospective studies and in particular the presence of HPVs prior to the development of HPV positive prostate cancer, (v) investigated the capacity of HPVs to transform normal prostate cells into malignant cells (vi) investigated the HPV oncogenic mechanisms in prostate cancer and (vii) considered the potential means of HPV transmission. In addition we conducted an ecological study in which we compared the mortality of presumed HPV associated cervical cancer with prostate cancer mortality in the same countries.

The main source of publications cited in this review is PubMed Central from 1980 to 2020 using the search terms prostate cancer and human papillomavirus. The references listed in these publications were also reviewed.

### Identification of human papillomaviruses in prostate tissues

Confirmation of the identity of a pathogen is an essential causal criterion.

HPV DNA (approximately 8 k base pairs) is circular, double stranded and is surrounded by a protein capsid coat. Eight genes are organised into early [E] and late [L]. E1 and E2 are involved with genome replication, transcription, segregation, encapsulation and apoptosis regulation of the cell. E4 is involved with cell cycle arrest and virion assembly. E5 controls cell growth and differentiation and modulates the immune system. E6 inhibits apoptosis and regulates cell signalling. E7 is involved with cell cycle control. L1 and L2 contribute to viral assembly. A persistent infection (one that is not cleared by the immune system) of high risk HPVs, can increase the risk of cancer via HPV oncogenes E6 and E7 which inactivate p53 and pRB. The disease progression of latent infection in the basal epithelial layer of organs such as the cervix, progresses from low-grade lesions, to later invasive cancer. This can take several years to decades.

Using standard polymerase chain reaction (PCR) technology and later Southern blots, McNichol et al. were the first to identify HPVs in prostate tissues [10]. Their findings have been confirmed in over 25 studies conducted in many different countries. High risk HPVs have since been identified in normal, benign and malignant prostate tissues as shown in Table 1. Virtually all of these studies have been based on standard PCR. PCR is susceptible to contamination leading to false outcomes. For this reason investigations based on alternative methods are of value. High risk HPVs in the nuclei of prostate cancers have been identified by in situ PCR [29]. In situ PCR is less liable to contamination than standard PCR. HPVs in prostate cancers have been identified by hybridisation methods [15, 38]. Oncogenic HPV E7 proteins have been identified in malignant prostate tissues [39, 40]. Using massive gene sequencing Glenn et al. identified high risk HPV types 16 and 18 in 12 of 502 invasive prostate cancers from The Cancer Genome Atlas [40]. Accordingly, it can be confirmed that high risk HPVs are present in normal, benign and malignant prostate tissues.

**Table 1:** Identification of high risk human papilloma viruses in prostate cancer. Case control studies with normal and benign prostate tissues as controls

Study	Country	Prostate cancer	Prostate control	Main HPV types	*P* value		
McNichol 1991 [12]	Canada	14/27 52%	1/5 20% normal 34/56 61% benign	16,18	0.396 ns 0.690 ns		
Anwar 1992 [14]	Japan	28/68 41%	0/10 0%	16,18,33	0.221 ns		
Ibrahim 1992 [15]	US	6/40 15%	2/29 7%	16	0.344 ns		
Rotola 1992 [16]	Italy	6/8 75%	14/17 82%	16	0.0.815 ns		
Dodd 1993 [17]	Canada	3/7 43%	5/10 50%	16	0.0.841 ns		
Tu 1994 [18]	US	1/43	0/1	16			
Moyret-Lalle 1995 [19]	France	9/17 53%	7/22 32%	16	0.682 ns		
Wideroff 1996 [20]	US	7/56 13%	4/42 10%	16,18,31,33,45	0.654 ns		
Terris 1997 [21]	US	10/53 19%	5/37 14% normal 7/21 33% benign	16 16	0.571 ns		
Serth 1999 [22]	Germany	10/47 21%	1/37 3%	16	0.027 s		
Carozzi 2004 [23]	Italy	6/24 25%	3/25 12%	16,18,31	0.333 ns		
Leiros 2005 [24]	Argentina	15/41 37%	0/30 0%	16	0.011 s		
Silvestre 2009 [25]	Brazil	2/65 3%	0/6 0%	16			
Martinez-Fierro 2010 [26]	Mexico	11/55 20%	4/75 5%	33,45,52,58,66	0.020 s		
Aghakhani 2011 [27]	Iran	10/104 10%	5/104 5%	16,18	0.213 ns		
Chen 2011 [12]	Australia	7/51 14%	3/11 27%	18	0.367 ns		
Tachezy 2012 [28]	Czech	1/51 2%	2/95 2%				
Whitaker 2013 [29]	Australia	29/50 58%	8/50 16%	18	0.003 s		
Ghasemian 2013 [30]	Iran	5/29 17%	8/167 5%		0.026 s		
Mokhtari 2013 [31]	Iran	3/30 10%	1/90 1%				
Michopoulou 2014 [32]	Greece	8/50 16%	1/30 3%	16, 18, 31	0.127 ns		
Singh 2015 [33]	India	36/95 38%	4/55 7%	16,18	0.001 s		
Huang 2016 [34]	China	30/75 40%	9/73 12%		0.001 s		
Davila Rodriquez 2016 [35]	Mexico	12/62 19%	1/25 4%	18,51,52	0.104 ns		
Atashafrooz 2016 [36]	Iran	16/100 16%	2/100 2%	16,18,31,33,54	0.002 s		
Medel Flores 2018 [37]	Mexico	37/189 20%	16/167 10%	16,18,31,33,52,58	0.014 s		

*p* = 0.05. s = significant. ns = not significant

### Strength and consistency of association between HPVs and prostate cancer

Consistency is considered to be an important causal criteria.

#### Case control studies

All published case control studies have been included. Therefore there is no selection bias. All studies which identified HPVs used PCR. The results are listed in Table 1. Studies in which high risk HPVs were not identified in prostate cancer tissues have also been included in Table 1. Twenty six case control studies were identified in which the prevalence of high risk HPVs in prostate cancers were compared to the prevalence in normal or benign prostate tissues. High risk HPVs were identified in 325 (22.6%) of 1437 prostate cancers and in 113 (8.6%) of 1313 normal or benign prostate controls (*p* = 0.001). Only one of the ten studies conducted before the year 2000 demonstrated a statistically significant difference between HPV positive benign and prostate cancer (94 of 366 prostate cancers [25.7%] and 80 of 287 benign prostate controls [27.9%] Compared to nine of 13 studies conducted after 2000 with 231 HPV positive of 1071 prostate cancers [21.6%] and 74 HPV positive of 1103 benign prostate controls [6.7%]). This reflects the increased quality of PCR analyses post 2000. These data are shown in Table 1. There are no differences in outcomes whether normal or benign prostate tissues were used as controls. HPVs were not identified in prostate cancers in 8 studies [41–48].

HPV types 16 and 18 which are known to be high risk for cancer, were the most commonly identified HPV types in these studies. However, in a number of studies these were the only HPV types sought to be identified by PCR primers.

The DNA sequence regions used in studies to identify HPV in prostate tissues are shown in Supplementary Table 2. There are predominantly two regions found by PCR namely L1, E6 /E7. The L1 region detects many different HPVs, the identity of which can be determined by hybridisation, line blots or sequencing or by using HPV kits. The L1 region is associated with viral assembly and not oncogenicity. The HPV E6 and E7 PCR primers can demonstrate that high risk for cancer HPV is present in prostate cancer or benign prostate tissues and is capable of producing oncogenic proteins.

Because of the conflicting outcomes of studies conducted in the same populations with both positive and negative identification of HPVs, the negative HPV identification may to be due to inadequate laboratory techniques. There are several reasons why research groups may have experienced difficulties when using PCR techniques for the identification of HPV gene sequences, (i) not all HPV PCR primers identify HPVs in prostate cancer, (ii) there is a low HPV viral load in prostate cancers as compared to the viral load in cervical cancer, (iii) fresh frozen samples give more consistent results than formalin fixed samples [21]. (iv) formalin fixed paraffin embedded DNA, after extraction, cannot always give a result if the PCR product is over 200 bp. The MY11/ MY9 primers from the L1 gene produce a 450 bp fragment [49].

#### In situ PCR

In situ PCR is conducted using formalin fixed tissue sections placed on glass slides. The risk of contamination is much less than standard liquid PCR. Using this method Whitaker et al. [29] identified high risk HPVs in 58% of 50 prostate cancers as compared to 16% of 50 benign prostate and 26% of normal prostate controls (*p* = 0.001). These outcomes were confirmed by standard PCR.

#### Immunohistochemistry

Oncogenic HPV E7 proteins have been identified by immunohistochemistry in 23 (82%) of 28 benign prostate specimens and 8 (29%) of 28 prostate cancers (*p* = 0.024) [39]. Using the same methods, this confirmed the identification of HPV E7 protein in 112 (75%) of 150 prostate cancers by Pascale et al. [39]. The high prevalence of HPV protein in benign prostate tissues suggests that HPV oncogenic influences may occur early in HPV associated prostate oncogenesis.

#### Whole genome sequencing

There are difficulties with the use of whole genome sequencing for the identification of HPVs in prostate cancer. HPV sequences were identified in only 2 of over 500 prostate cancer specimens from The Cancer Genome Atlas (TCGA) by Tang et al. as compared to the frequent identification by PCR techniques of HPV gene sequences in up to 75% of prostate cancers [50]. On the other hand, Glenn et al. used whole genome sequencing on the same TCGA data based on the same prostate cancer specimens and identified HPV types 16 and 18 in 17 of 503 prostate cancer specimens [40]. Despite the low identification of high risk HPVs in the TCGA prostate cancer series, the outcomes confirm their identification by PCR and immunohistochemistry [40]. The reasons for the low identification of HPVs by whole genome sequencing have been considered by Vinner et al. who have shown that these techniques are unlikely to detect viruses with very low concentrations in cancers as compared to amplification techniques such as PCR [51]. Vinner et al. argue that despite the enormous data output from massive parallel sequencing, viral DNA in a clinical sample typically constitutes a proportion of host DNA that is too small to be detected [51]. In addition viruses need to be integrated into the host genome to enable identification by massive parallel sequencing.

#### Serology

All published studies based on serology have been included. Therefore there is no selection bias. In 14 different studies HPV antibodies were identified in the serum of 1019 (20%) of 5149 men with prostate cancer as compared to 1546 (20%) of 7794 normal controls (Table 2). Overall, there is no difference between the prevalence of HPV antibodies in the sera of men with or without prostate cancer. The prevalence of HPV antibodies is present in up to 68% of the serum of both men with prostate cancer and normal controls (Table 2). Although there are statistically significant differences in many of these studies between the prevalence of HPV antibodies in the serum of patients with prostate cancer as compared to normal controls, these differences are very small. This is in contrast to the prevalence of HPV serum antibodies in patients with HPV associated cervical cancer which are much higher than in normal controls [65].

**Table 2:** Identification of antibodies to high risk human papilloma viruses in prostate cancer. Case control serological studies with normal sera as controls

Study	Country	Prostate cancer	Controls	Main HPV types	P value
Dillner 1998 [52]	Finland	21/165 13%	36/290 12%	16,18,33	0.865 ns
Hisada 2000 [53]	US	20/48 42%	19/63 30%	16	0.253 ns
Hayes 2000 [54]	US	19/276 7%	15/295 5%	16	0.393 ns
Rosenblatt 2003 [55]	US	81/642 13%	64/570 11%	16,18	0.347 ns
Adami 2003 [56]	Sweden	69/238 29%	48/210 23%	16,18,33	0.212 ns
Korodi 2005 [57]	Sweden	107/799 13%	363/2596 14%	16,18,33	0.482 ns
Sutcliffe 2007 [58]	US	107/584 18%	114/577 20%	16,18,33	0.696 ns
Sitas 2007 [59]	South Africa	139/205 68%	390/673 58%	16	0.001 s
Huang 2008 [60]	US	154/868 18%	310/1283 24%	16,18	−0.002 s
Dennis 2009 [61]	US	50/267 19%	45/267 17%		0.637 ns
Sutcliffe 2010 [62]	US	23/616 4%	22/616 4%	16,18,31	0.686 ns
Chen 2011 [12]	Australia	29/53 55%	41/104 39%	16,18,31,33,52,58	0.131 ns
Hrbacek 2011 [63]	Czech	167/316 53%	69/101 68%	16,18,31,33	−0.161 ns
Tachezy 2012 [28]	Czech	14/50 28%	37/173 22%	16,18,31,33	0.445 ns
Zhao 2017 [64]	China	48/75 64%	14/80 17.5%	16	0.001 s

s = significant at *p* = 0.05. ns = not significant

Of the 15 serology studies in Table 2, only Chen et al. [12] and Tachezy et al. [28] used PCR on prostate cancer tissues. The PCR was not matched with the serology in either study.

Zhao et al. have used a new technique “seroscreening by microarray” to identify HPV antibodies in serum samples [64]. They identified HPV type 16 antibodies in 48 (64%) of 75 serum samples from men with prostate cancer and 14 (17.5%) of 80 normal controls (*p* = 0.001).

The implication of these observations is not clear because serology identifies overall exposure to HPV infections but does not identify HPV infections of specific organs such as the prostate.

#### Prospective studies

Glenn et al. identified high risk HPVs in benign prostate tissues from patients who 1 to 10 years later developed HPV positive prostate cancer of the same type [40]. The same HPV types were present in both the benign and subsequent prostate cancers in 9 sets of specimens.

These findings can be interpreted as evidence of persistent infection of the prostate by HPVs.

### Transmission

Information about transmission of a pathogen can be a helpful causal criteria. This information can also be used to develop preventive strategies.

Sexually transmitted human papillomavirus infections increase the risk of prostate cancer by up to 40% [66]. HPV DNA is detectable in urine of a high proportion of the sexually active British population [67]. In both genders, high risk HPV is strongly associated with sexual behaviour. The most likely transmission of HPVs is during sexual activities by cell surface to surface contact.

Based on a meta-analysis of 31 studies conducted in a wide range of countries, high risk HPVs are present in semen in approximately 10% of the general population [68]. HPV 16 is the most common type in semen. As HPV 16 is also the most common type identified in prostate cancers it is likely that HPVs can be transmitted from semen to the prostate.

There is recent evidence that HPVs can be transmitted throughout the body via circulating extra – cellular vesicles [69, 70]. Extra – cellular vesicles are released from different types of tissue, cells and biological fluids and contain nucleic acids, proteins, non-coding RNAs and viral nucleic acids. Exosomes and extracellular vesicles have been implicated in HPV transmission and carcinogenesis [69]. HPV’s can also be distributed throughout the body in circulating blood. Gupta et al. have shown that in healthy blood donors from Qatar, (98% of which were male) 47% had high risk HPV’s [71].

### Epidemiology

High risk HPV infections are associated with up to 99% of cervical cancers. As both cervical and prostate cancer are associated with HPV sexually transmitted infections, it is relevant that there is a positive correlation between cervical cancer mortality and prostate cancer mortality. High cervical cancer mortality rates are correlated with high prostate cancer mortality rates. Low cervical cancer mortality rates are correlated with low prostate cancer mortality rates. This is shown for selected countries in Table 3. This is also shown for the 77 countries for which data is available in Supplementary Table 1. The correlation is statistically significant (*p* = 0.001). These data are associations and do not offer conclusive evidence that HPVs are causal in prostate cancer. The data is however, compatible with the hypothesis that HPVs may have a causal role in prostate cancer.

**Table 3:** Cervical cancer and prostate cancer death rates per 100,000 population age adjusted for 16 selected countries 2015–2018. Cervical and prostate cancer death rates correlate. Source: World Health Organisation International Agency for Research on Cancer 2019

Country	Cervical cancer	Prostate cancer
South Korea	1.9	4.5
China Hong Kong	1.9	4.5
Japan	2.0	4.8
Italy	0.7	7.4
United States America	1.7	8.4
France	1.3	9.1
Germany	1.8	11.3
Australia	1.2	11.4
Brazil	4.5	14.5
Chile	4.6	15.2
South Africa	12.2	18.3
Suriname	12.4	19.6
Venezuela	9.3	20.3
Cuba	5.0	23.7
Barbados	10.5	34.5
Trinidad Tobago	8.5	37.8

This is illustrated by the South African experience. Among South African men HPV antibody levels are very high in the serum from men with prostate cancer (68%) and in benign prostate controls (58%) [59]. The mortality rates of both cervical and prostate cancer in South Africa are high (cervical cancer 12.2, prostate cancer 18.3 per 100,000 of the South African population as compared to 1.9 and 4.5 respectively for the South Korean population - Table 3). The likely explanation is that high levels of unprotected sexual activity in South Africa has led to high levels of sexually transmitted infections which include high risk HPVs.

### Transformation and causal mechanisms

Information about transformation and causal mechanisms have been added to the original Hill causal criteria.

Exposure of cultured normal and benign prostate epithelial cells to HPV 16 and 18 induces immortalisation of these cells [72–75]. Schutze et al. have experimentally demonstrated the immortalisation capacity of 11 different HPV types [76]. This capacity varies according to the HPV type. This immortalisation, seen initially in cervical cells, is not confined to prostate epithelial cells. Oral epithelial cells, human embryonic fibroblasts and primary human keratinocytes can also be immortalised.

The joint action of E6 and E7 oncoproteins target cellular pathways which involve cell cycle control and apoptosis and thus enable cell proliferation. Upon integration these genes drive cellular immortalisation. E5 can cooperate with E6 and E7 enhancing transformation activity in precancerous lesions [77].

Immortalised benign prostate cultured cells can acquire neoplastic properties when further exposed to other viruses, such as the murine sarcoma virus and also components of HPVs such as lipopolysaccharides [72, 77].

The oncogenic influences of high risk HPVs in prostate cancer may be both direct and indirect. The evidence for a direct role of HPVs in prostate cancer includes the presence of HPV related koilocytes in both benign and malignant prostate cancer tissues [29, 37]. Koilocytes are large cells with perinuclear halos. HPV associated koilocytes are associated with the activity of low and high risk HPV E5 and E6 proteins [78]. The appearance of koilocytes is an early sign of HPV infections and early oncogenesis of the cervix. Glenn et al. have shown that the oncogenic protein HPV E7 is strongly expressed in HPV positive benign prostate tissues but 1 to 11 years later are weakly or not expressed in HPV positive prostate cancer tissues in the same patients [40]. These observations suggest that HPVs may have early but not necessarily continuing direct oncogenic influences on prostate tissues.

The evidence for an indirect role of HPVs in prostate cancer is the observation that the incidence of prostate cancer falls by up to 30% in immunocompromised men whereas the incidence of cervical cancer increases threefold in immunocompromised women [79]. These incidence patterns are similar when the immunocompromise is due to either human immunodeficiency disease or transplantation. Grulich and Vladich argue that these observations indicate that infections do not have a role in prostate cancer. In our view this is not correct as HPV oncogenic influences may be indirect [79]. This view is based on evidence that HPV infections can inhibit the protective role of APOBEC enzymes [80, 81]. These enzymes have evolved as protection from viral infections. Significant APOBEC mutations have been observed in prostate cancers [82].

The changed characteristics of APOBEC3B reduces its protective effects against oncogenic viruses [83]. The main mechanism with respect to HPVs and prostate cancer is probably indirect and involves the influence of HPVs on enzymes such as APOBEC3B which usually help protect against the harmful effect of viruses [80, 81]. After HPV viral DNA integration, mutations in APOBEC3B can lead to host genome instability and then to cancer progression [80, 81]. A further complexity is the apparent collaboration between HPVs and EBV and the adverse influence of EBVs on the integrity of APOBEC 52]. Epstein Barr virus has been identified in prostate cancer [29]. Gupta et al. has also identified EBV (61%) in the blood of healthy donors [71].

### Specificity

This original Hill criteria has lapsed because it is now known that specific pathogens can infect multiple organs. Infections with high risk HPVs identified in multiple organs is a good example.

High risk HPVs have causal roles in both cervical cancer and head and neck cancers. High risk HPVs have also been identified in a wide range of cancers including breast, anal, penile, vulva, vaginal, and colorectal cancer [84, 85]. Accordingly HPVs are not specific for prostate cancer.

### Temporality

HPV infection and later development of the same virus positive prostate cancer in the same patient. This is a valuable Hill causal criteria.

High risk HPVs are present in benign prostate tissues prior to the development of the same type HPV positive prostate cancer in the same patients [40]. Dodd et al. have demonstrated that HPV E6/E7 viral gene transcripts can be present in both benign and malignant prostate tissues [17]. In addition as shown in Table 1 high risk HPVs have been identified in benign prostate tissues as well as prostate cancers in many studies.

As referred to above, there is a significantly higher expression of HPV E7 oncoproteins in benign prostate tissues as compared to late prostate cancer that subsequently developed in the same patients [40]. This observation suggests that HPV oncogenic activity is an early phenomenon in a majority of prostate oncogenesis.

### Biological gradient

This original Hill criteria implied that an increased pathogen load should lead to increased pathology. This is not an important criteria as it is now known that with progression of cancer, the physiology of cells can break down and the pathogen load may decrease or even disappear.

On the other hand in a study of Japanese men with prostate cancer, Anwar et al. observed an increased prevalence of HPV infections in advanced stages of prostate cancer [14]. In a more recent study of Indian men with prostate cancer, Singh et al. observed a significant increase in prevalence of HPV infections in late stage prostate cancers as compared to benign and early stage prostate cancer tissues [33].

### Plausibility, analogy

This original Hill criteria remains useful because it is now known that an infectious pathogen may attack multiple organs. Therefore if there is evidence of HPV associated oncogenicity as is the case of HPV and cervical cancer, it is plausible that HPVs can initiate oncogenicity in other organs. Accordingly it is plausible that HPVs may have an oncogenic capacity to cause prostate cancers.

### Multiple pathogenic agents

As outlined above, multiple pathogens including both viruses and bacteria have been identified in prostate cancer tissues [2–4, 29]. These pathogens include human papillomaviruses, Epstein Barr virus and commonly *Propionibacterium acnes*. HPV and Epstein Barr viruses can co-exist on normal, benign and malignant prostate tissues [29]. There is experimental evidence that Epstein Barr virus can enhance the oncogenicity of HPVs [86].

### Human papillomavirus, prostatitis, benign prostate hyperplasia, prostate cancer

Based on meta-analyses of 21 studies by Zhang et al., there is a correlation and enhanced risk between prostatitis leading to benign prostate hyperplasia and finally prostate cancer [87]. Multiple pathogenic organisms including high risk HPVs have been identified in prostatic secretions and semen of patients with prostatitis [88–91]. The biology underlying inflammation and cancer is complex particularly if infectious agents such as HPVs are involved. Benign prostate tissues cannot be considered as normal and inflammatory prostatitis may develop into benign prostate hyperplasia and prostate cancer. These issues are further complicated because of the adverse influences of HPVs on the antiviral role of the APOBEC family of enzymes.

### Prevention

There are three preventative vaccines against HPV infections, Cervarix®. protects against HPV 16 and 18, Gardasil® protects against HPV 16,18, 6 and 11, Gardasil® 9 protects against HPV 6,11,16,18, 31,33,45,52,and 58. (HPVs 6 and 11 cause genital warts) Gardasil and Cervarix, have been widely used during the past decade [92], but Gardasil® 9 is the current choice.. These vaccines have been shown to be both effective and safe. Their early use was mainly in young girls to prevent cervical cancer. Their use has been recommended to boys to prevent genital warts and penile cancer. It is not known how long HPV vaccines administered to young males can remain effective. This is relevant to prostate cancer which may not develop for decades after an initial infection by HPVs.

## Discussion and conclusions

The evidence for a causal role of high risk HPVs in prostate cancer is as follows:

(i) High risk for cancer HPVs have been identified by a range of methods in normal, benign and malignant prostate tissues in a wide range of countries [9, 12, 15, 29].
(ii) In 8 of 26 case control studies the prevalence of high risk HPV DNA was significantly higher in prostate cancers as compared to normal and benign prostate controls. In the more recent studies conducted after the year 2000 and using increasingly sophisticated PCR techniques, there were 231 HPV positive of 1071 prostate cancers [21.6%] and 74 HPV positive of 1103 benign prostate controls [6.7%] (*p* = 0.001).
(iii) HPV antibodies have been identified in the blood serum of both normal men and prostate cancer patients.
(iv) High risk HPVs have been identified in benign prostate tissues 1 to 11 years before the development of HPV positive (same type as prior benign) prostate cancer in the same patients [40].
(v) High risk HPV types 16 and 18 have the capacity to immortalise and transform normal prostate cells into malignant cells [72–76].
(vi) The most frequent means of HPV transmission is probably by sexual activity [21, 47, 48]. In addition, HPVs can be transmitted throughout the body via circulating extra – cellular vesicles and blood [69–71].
(vii) The oncogenic mechanisms for HPV oncogenesis in prostate cancer are not clear. There is evidence that HPV E7 oncogenic proteins may be directly involved early in oncogenesis [40]. HPV infections may have an indirect role by inhibiting the protective function of APOBEC3B enzymes against other virus infections [80, 81].
(viii) There is evidence that high risk HPVs can be associated with inflammatory prostatitis which can lead to benign prostate hyperplasia and later prostate cancer [87–91].
(ix) There is ecological evidence which suggests that HPVs may have causal roles in both prostate and cervical cancer in the same populations.

The most important evidence is the reasonably consistent evidence that high risk HPVs are significantly more prevalent in prostate cancers than in normal prostate tissues and benign prostate tissues. Evidence of changes leading to cancer, were demonstrated by Glenn et al., who showed that (i) high risk HPVs are present in benign prostate tissues who up to 10 years later developed HPV positive prostate cancer of the same HPV type in the same patients, (ii) HPV E7 oncoprotein was much more highly expressed in benign prostate tissues as compared to prostate cancer which had developed years later in the same patients thus indicating early HPV related oncogenesis, (iii) identification of high risk HPV RNA sequence data by massive parallel sequencing indicates that some prostate HPV cancers still have biologically active oncoproteins [40].

The additional evidence, namely, temporality, biological gradient, immortalisation by HPVs and sexual transmission of HPVs, give added plausibility and coherence of HPV’s contributing to prostate cancer.

However, sound evidence regarding HPV oncogenic mechanisms in prostate cancer is missing. To find this evidence, genome studies using HPV introduction into normal prostate cells may elucidate the mechanisms involved. This could best be achieved using human cells, in tissue culture. Animal cells and models could also be used, but each papillomavirus is host specific. Growing human papilloma virus (HPV) in nude mice transplanted with human prostate tissue could be another alternative.

The role of HPVs in prostate cancer is complex and differs from HPVs associated cervical cancer. HPV infections may initiate prostate oncogenesis directly and influence oncogenesis indirectly via APOBEC enzymes. In addition HPVs may collaborate with other pathogens in prostate oncogenesis.

## Conclusions

We have used the term “highly likely” because it is less dogmatic than “conclusive”.

Overall, the evidence that high risk HPVs have oncogenic roles in prostate cancer meets the key Bradford Hill causal criteria. It is “highly likely” that high risk HPVs have causal roles in prostate cancer. This evidence is sufficiently sound to justify its use in encouraging universal vaccination against HPV infections.

## Supplementary information

**Additional file 1 Supplementary Table 1.** Cervical cancer and prostate cancer death rates per 100,000 population age adjusted for 77 countries 2015–2018. Source: World Health Organisation International Agency for Research on Cancer 2019.Up to 99% of cervical cancers are human papillomavirus related. There is a significant correlation between death rates for cervical cancer and prostate cancer in the 77 countries for which data is available. Pearson correlation = 0.437; *p* = 0.001

**Additional file 2 Supplementary Table 2.** PCR primers used for the detection of HPV DNA. The L1 gene primers are usually MY11 to MY9 (sometimes followed by a nested Gp5+ to Gp6+), or FAP which amplify different types of HPV’s, such as, low risk types 6,11,and high risk types 16,18 and 33. The 450 bp products were initially typed by hybridisation or line blots using HPV specific probes, but this typing was later replaced by sequencing. Positive results for the L1 region only confirm the presence of HPV DNA. The E6 and or E7 primers however can relate to oncogenicity of HPV in the tissues

## Data Availability

All data relevant to this manuscript is available in this article.

## Abbreviations

APOBEC: Apolipoprotein B mRNA editing enzyme, catalytic polypeptide-like
HPV: Human papillomavirus
PCR: Polymerase chain reaction
