# Supplementary material for: Evidence for a causal role by human papillomaviruses in prostate cancer – a systematic review
Source: Infect Agent Cancer. 2020 Jul 14;15:41. doi: 10.1186/s13027-020-00305-8 (PMC7359253; doi:10.1186/s13027-020-00305-8)
Supplement: Supplementary file 1 — Additional file 1 Supplementary Table 1. Cervical cancer and prostate cancer death rates per 100,000 population age adjusted for 77 countries 2015–2018. Source: World Health Organisation International Agency for Research on Cancer 2019.Up to 99% of cervical cancers are human papillomavirus related. There is a significant correlation between death rates for cervical cancer and prostate cancer in the 77 countries for which data is available. Pearson correlation = 0.437; p = 0.001 [file 13027_2020_305_MOESM1_ESM.docx]

| **Country** | **Cervical cancer** | **Prostate cancer** |
| --- | --- | --- |
| Albania | 0.7 | 5.7 |
| Argentina | 4.5 | 12.8 |
| Armenia | 2.6 | 12.0 |
| Australia | 1.2 | 11.4 |
| Austria | 1.6 | 10.0 |
| Azerbaijan | 1.6 | 3.3 |
| Barbados | 10.5 | 34.5 |
| Belarus | 4.1 | 12.1 |
| Belgium | 1.3 | 9.3 |
| Belize | 12.0 | 16.4 |
| Brazil | 4.5 | 14.5 |
| Bulgaria | 5.7 | 10.9 |
| Canada | 1.4 | 9.4 |
| Chile | 4.6 | 15.2 |
| China Hong Kong | 1.9 | 4.5 |
| Columbia | 5.7 | 11.2 |
| Costa Rica | 4.2 | 13.2 |
| Croatia | 2.6 | 14.6 |
| Cuba | 5.0 | 23.7 |
| Cyprus | 1.8 | 9.2 |
| Denmark | 1.7 | 15.3 |
| Dominican Republic | 4.4 | 16.6 |
| Ecuador | 0.7 | 11.2 |
| Egypt | 0.4 | 2.8 |
| El Salvador | 5.6 | 7.4 |
| Estonia | 4.4 | 19.5 |
| Finland | 1.1 | 11.2 |
| France | 1.3 | 9.1 |
| Georgia | 4.8 | 8.5 |
| Germany | 1.8 | 11.3 |
| Greece | 1.5 | 8.2 |
| Guatemala | 6.5 | 9.7 |
| Hungary | 4.0 | 13.7 |
| Iceland | 3.1 | 12.1 |
| Ireland | 2.6 | 12.3 |
| Israel | 1.5 | 5.8 |
| Italy | 0.7 | 7.4 |
| Japan | 2.0 | 4.8 |
| Kazakhstan | 5.7 | 5.8 |
| Kuwait | 0.7 | 10.1 |
| Kyrgystan | 8.1 | 4.0 |
| Latvia | 6.0 | 19.9 |
| Lithuania | 6.6 | 17.3 |
| Luxemburg | 0.9 | 8.5 |
| Malta | 1.4 | 7.9 |
| Mauritus | 4.2 | 8.9 |
| Mexico | 5.9 | 10.8 |
| New Zealand | 1.6 | 13.0 |
| Nicaragua | 9.4 | 9.1 |
| North Macedonia | 2.4 | 14.2 |
| Norway | 2.0 | 14.2 |
| Panama | 6.1 | 11.8 |
| Paraguay | 9.6 | 12.6 |
| Peru | 6.0 | 9.4 |
| Philippines | 4.6 | 11.2 |
| Poland | 4.1 | 13.7 |
| Portugal | 1.7 | 11.3 |
| Maldova | 5.4 | 10.9 |
| Romania | 8.4 | 10.3 |
| Russian Federation | 5.3 | 11.3 |
| Serbia | 6.7 | 10.9 |
| Singapore | 2.3 | 5.6 |
| Slovakia | 4.8 | 16.8 |
| South Korea | 1.9 | 4.5 |
| South Africa | 12.2 | 18.3 |
| Spain | 1.4 | 8.6 |
| Suriname | 12.4 | 19.6 |
| Sweden | 1.7 | 15.1 |
| Switzerland | 1.1 | 10.4 |
| Tajikistan | 3.2 | 1.4 |
| Thailand | 5.2 | 2.3 |
| The Netherlands | 1.4 | 12.4 |
| Trinidad Tobago | 8.5 | 37.8 |
| Turkmenistan | 5.3 | 2.2 |
| Ukraine | 4.9 | 10.4 |
| United Kingdom | 1.6 | 12.8 |
| Uruguay | 3.1 | 17.7 |
| United States America | 1.7 | 8.4 |
| Uzbekistan | 5.1 | 2.2 |
| Venezuela | 9.3 | 20.3 |
|  |  |  |

**Supplementary Table 1. Cervical cancer and prostate cancer death rates per 100,000 population age adjusted for 77 countries 2015 -2018.** Source: World Health Organisation International Agency for Research on Cancer 2019.

Up to 99% of cervical cancers are human papilloma virus related. There is a significant correlation between death rates for cervical cancer and prostate cancer in the 77 countries for which data is available. Pearson correlation = 0.437; p = 0.001
