# Supplementary material for: Evidence for a causal role by human papillomaviruses in prostate cancer – a systematic review
Source: Infect Agent Cancer. 2020 Jul 14;15:41. doi: 10.1186/s13027-020-00305-8 (PMC7359253; doi:10.1186/s13027-020-00305-8)
Supplement: Supplementary file 2 — Additional file 2 Supplementary Table 2. PCR primers used for the detection of HPV DNA. The L1 gene primers are usually MY11 to MY9 (sometimes followed by a nested Gp5+ to Gp6+), or FAP which amplify different types of HPV’s, such as, low risk types 6,11,and high risk types 16,18 and 33. The 450 bp products were initially typed by hybridisation or line blots using HPV specific probes, but this typing was later replaced by sequencing. Positive results for the L1 region only confirm the presence of HPV DNA. The E6 and or E7 primers however can relate to oncogenicity of HPV in the tissues [file 13027_2020_305_MOESM2_ESM.docx]

| **Study** | **Reference** | **Gene Detected** |
| --- | --- | --- |
| McNichol 1991 | [10] | E6 |
| Anwar 1992 | [57] | E6 |
| Ibrahim 1992 | [15] | L1 |
| Rotola 1992 | [66] | *E6 |
| Dodd 1993 | [56] | *E6/E7 |
| Tu 1994 | [67] | L1 |
| Moyret-Lalle 1995 | [68] | E6 |
| Wideroff 1996 | [69] | L1 |
| Terris 1997 | [27] | *L1, E6 x 2 |
| Serth 1999 | [70] | *E6 |
| Carozzi 2004 | [71] | L1, E6/E7 |
| Leiros 2005] | [72] | L1 |
| Silvestre 2009 ] | [73] | L1 |
| Martinez-Fierro 2010 | [74] | L1 |
| Aghakhani 2011 | [75] | L1 |
| Chen 2011 | [12] | L1 |
| Tachezy 2012 | [32] | L1 |
| Whitaker 2013 | [14] | L1 |
| Ghasemian 2013 | [76] | L1 |
| Mokhtari 2013[ | [77] | IHC |
| Michopoulou 2014 | [78] | L1 |
| Singh 2015 | [58] | L1 |
| Huang 2016 | [79] | ND |
| Davila Rodriquez 2016 | [80] | L1 |
| Atashafrooz 2016 | [81] | ND |
| Medel Flores 2018 | [47] | E6/E7 |

**Supplementary Table 2. PCR primers used for the detection of HPV DNA.**

The L1 gene primers are usually MY9 to MY11 monoclonal antibodies (sometimes followed by a nested Gp5+ to Gp6+), or FAP which amplify different types of HPV's, such as, low risk types 6,11,and high risk types 16,18 and 33. The 450 bp products were initially typed by hybridisation or line blots using HPV specific probes, but this typing was later replaced by sequencing. Positive results for the L1 region only confirm the presence of HPV DNA. The E6 and or E7 primers however can relate to oncogenicity of HPV in the tissues. * In some studies only HPV16 PCR primers were used. ND = not determined.
